# Interleukin-Mediated Pendrin Transcriptional Regulation in Airway and Esophageal Epithelia

**DOI:** 10.3390/ijms20030731

**Published:** 2019-02-09

**Authors:** Simone Vanoni, Giada Scantamburlo, Silvia Dossena, Markus Paulmichl, Charity Nofziger

**Affiliations:** 1 Pharmgenetix GmbH, A-5081 Niederalm, Austria; giada.scantamburlo@pharmgenetix.com (G.S.); charity.nofziger@pharmgenetix.com (C.N.); 2 Institute of Pharmacology and Toxicology, Paracelsus Medical University, A-5020 Salzburg, Austria; silvia.dossena@pmu.ac.at; 3 Department of Personalized Medicine, Humanomed, A-9020 Klagenfurt, Austria; paulmichl@me.com

**Keywords:** pendrin, interleukins, airway epithelium, esophageal epithelium, asthma, eosinophilic esophagitis

## Abstract

Pendrin (SLC26A4), a Cl^−^/anion exchanger, is expressed at high levels in kidney, thyroid, and inner ear epithelia, where it has an essential role in bicarbonate secretion/chloride reabsorption, iodide accumulation, and endolymph ion balance, respectively. Pendrin is expressed at lower levels in other tissues, such as airways and esophageal epithelia, where it is transcriptionally regulated by the inflammatory cytokines interleukin (IL)-4 and IL-13 through a signal transducer and activator of transcription 6 (STAT6)-mediated pathway. In the airway epithelium, increased pendrin expression during inflammatory diseases leads to imbalances in airway surface liquid thickness and mucin release, while, in the esophageal epithelium, dysregulated pendrin expression is supposed to impact the intracellular pH regulation system. In this review, we discuss some of the recent findings on interleukin-mediated transcriptional regulation of pendrin and how this dysregulation impacts airway and esophagus epithelial homeostasis during inflammatory diseases.

## 1. Introduction

Pendrin (SLC26A4) is an electroneutral anion exchanger, transporting iodide, bicarbonate, hydroxide, thiocyanate, and formate for chloride [1]. It is a 780-amino-acid-long highly hydrophobic glycoprotein [2], with three putative extracellular glycosylation sites [3,4]. Similar to the other SLC26 family members, pendrin’s structure is composed of 14 membrane-spanning α-helices forming the N-terminal transmembrane domain (TMD) connected to a C-terminal cytosolic sulfate transporter anti-sigma factor antagonist (STAS) domain [5,6]. Since its identification in 1997 as the gene causing Pendred syndrome [2], an autosomal recessive disorder leading to sensorineural hearing loss and an enlarged thyroid, pendrin was observed to be highly expressed in epithelial cells of the inner ear [7], thyroid gland [2], and kidney [8]. In the inner ear, pendrin mediates Cl^−^/HCO_3_^−^ exchange in order to control the acid–base balance of the endolymph, an essential requisite for a normal hearing [9,10,11]. In the thyroid gland, pendrin action is fundamental for iodide efflux into the follicular lumen, by exchanging Cl^−^ for I^−^ [12,13], while, in the kidney, pendrin contributes to acid–base balance and the regulation of blood pressure at the level of the cortical collecting duct, by secreting HCO_3_^−^ into the tubular lumen in exchange of Cl^−^ [14,15,16,17]. Baseline levels of pendrin were also shown in other tissues, such as the airway [18,19,20,21,22,23], mammary gland [24], testis [25], placenta [26], endometrium [27], liver [28], and esophageal epithelia [29]. Regarding the airways and the esophageal epithelia, the role and expression of pendrin become evident when studied in correlation with specific diseases such as asthma, chronic obstructive pulmonary disease (COPD), and eosinophilic esophagitis (EE). An upregulation of pendrin expression was indeed demonstrated in the aforementioned pathologies, and such an upregulation is predominantly mediated by interleukin (IL)-4, IL-13 and IL-17 [19,20,21,30]. In this review, attention is focused on interleukin-mediated transcriptional regulation of pendrin in bronchial and esophageal epithelia, its effects on the physiology of these two tissues, and the relationship with the etiology of particular diseases.

## 2. The Interleukins

A common feature of the inflammatory diseases, among others, is the intense release and accumulation of cytokines at the inflamed site [31]. When considering the pathophysiology of respiratory diseases such as asthma or COPD, interleukins, especially IL-4, IL-13, IL-1β, and IL-17, are the major inflammatory instigators [32,33,34,35]. Similarly, EE is a typical T helper cell type 2 (Th2) disease and also driven by the release of IL-13 and IL-5 [36,37]. IL-4 is a cytokine fundamental for the differentiation of naive cluster of differentiation 4^+^ (CD4^+^) T cells into Th2 cells [38], and is initially produced by mast cells, eosinophils, and basophils [39,40,41]. Following differentiation, Th2 cells activate a positive feedback loop to produce further IL-4 and recruit macrophages, basophils, and eosinophils into the inflammatory zone [42]. Homologous to IL-4, IL-13 is another cytokine mainly secreted by Th2 cells, although it can also be produced by natural killer T (NKT) cells, mast cells, basophils, eosinophils, and nuocytes [43]. Two receptors on the surface of target cells are able to recognize IL-4, and both have in common the IL4Rα subunit. In hematopoietic cells, the IL4Rα chain pairs with the IL-2Rγ chain to form the type I receptor [44,45,46], while, in non-hematopoietic cells, the IL4Rα chain instead pairs with the IL13Rα1 chain forming the type II receptor [47]. IL-13, in contrast, can only be recognized by the type II receptor and is also able to engage a decoy receptor, formed by the IL13Rα2 chain [48]. Upon binding of their respective receptors, both IL-4 and IL-13 activate the Janus kinase (JAK)/signal transducer and activator of transcription (STAT) signaling pathway. STAT6 is the major transcription factor activated by this cascade through phosphorylation of its tyrosine residue number 641 [49]. Once phosphorylated, STAT6 dimerizes in the cytoplasmic region via its Src homology 2 (SH2)-domain and, afterward, translocates into the nucleus where it binds specific consensus DNA motifs [50]. The preferred DNA-binding site for STAT6 is a palindromic sequence 5′ TTC (N_4_) GAA 3′ (where N is any nucleotide), otherwise known as the N_4_ gamma activated sequence (GAS) motif [51]. Nevertheless, some studies demonstrated that, albeit with lower frequency, STAT6 can also bind the 5′ TTC (N_3_) GAA 3′ motif, similarly to other members of the STAT family [52,53,54]. Several studies aiming to define the role of STAT6 in allergic asthma highlighted its importance in this pathological condition, since mouse models constitutively expressing STAT6 are prone to allergic phenotypes [55], while, in contrast, in vivo models lacking this transcription factor are protected from allergy [56,57]. Interestingly, STAT6 can cooperate with other transcription factors, such as nuclear factor kappa-light-chain-enhancer of activated B cells (NF-κB) [58], CCAAT/enhancer-binding protein β (C/EBPβ) [59], cAMP response element-binding (CREB)-binding protein (CBP) [60], and steroid nuclear receptor co-activator (NcoA-1) [61].

IL-17 is strictly associated with allergic responses inducing proinflammatory gene expression [62,63], acting alone or in combination with tumor necrosis factor-α (TNF-α), IL-6, Granulocyte colony-stimulating factor (G-CSF), IL-1, chemokine (C-X-C motif) ligand 1 (CXCL1), chemokine (C-C motif) ligand 20 (CCL20), and matrix metalloproteinases [64,65,66] through the activation of NF-κB, mitogen-activated protein kinase (MAPK), and C/EBP cascades [67]. Of note, IL-17 was also shown to activate the JAK/STAT and the JAK/phosphoinositide-3-kinase (PI3K) pathways in human airway epithelial and smooth muscle cells [68,69]. Moreover, several studies demonstrated the ability of IL-17 to stabilize the messenger RNA (mRNA) of pro-inflammatory genes by dissociating mRNA splicing factors, thereby prolonging their half-lives and avoiding consequent degradation [70,71]. This interleukin is mainly produced by Th17 cells [72], but its expression can also be detected in eosinophils [73], neutrophils [74], monocytes [75], macrophages [76,77], and lymphocytes [78,79,80]. Of particular interest is that IL-17 was shown to be upregulated in patients with asthma [73], chronic rhinosinusitis [81], and COPD [82].

Secreted by alveolar macrophages and peripheral blood mononuclear cells, IL-1β is one of the most important cytokines behind the initiation and persistence of inflammation [83], especially with regards to COPD exacerbation [84]. Indeed, IL-1β is sufficient to induce symptoms such as emphysema, neutrophil and macrophage infiltration, airway fibrosis, lymphocytic nodules, and mucous cell hyperplasia, all characteristics of COPD or chronic asthma [85,86]. In response to binding of IL-1β to the IL-1 receptor (IL-1R), a complex signaling cascade involving (i) the phosphorylation of the IL-1 receptor-activated protein kinase 4 (IRAK4), (ii) the phosphorylation of the ubiquitine ligase tumor necrosis factor receptor-associated factor 6 (TRAF6), (iii) the ubiquitination of the transforming growth factor-β (TGF-β)-activated protein kinase1 (TAK1), and (iv) its association with the mitogen-activated protein kinase kinase kinase 3 (MEKK3), leads to the activation of several transcription factors, such as NF-κB, c-Jun N-terminal kinase (JNK), and p38 MAPK) [87]. As a result, the expression of multiple genes, such as IL-6, IL-8, monocyte chemoattractant protein-1 (MCP-1) and cyclooxygenase-2 (COX-2) [87], ensues.

## 3. Pendrin in the Airways

The involvement of pendrin in airway diseases was first reported in 2005 [19]. Since then, the exchanger was shown to be upregulated in airway epithelial cells following stimulation with allergic cytokines such as IL-13, IL-4, and IL-17, in asthma or COPD mouse models and in patients with asthma, cystic fibrosis (CF), rhinovirus infections, rhinitis, chronic rhinosinusitis, and pertussis infection [19,20,21,30,88,89,90,91,92,93]., In particular, the pathogenesis and disease severity of asthma, allergic rhinitis, and chronic sinusitis with nasal polyps are driven by the activation of eosinophils and CD4^+^ cells, leading to a Th2 cytokine response which mainly includes IL-4 and IL-13 release [42,94,95]. In addition to these two cytokines, IL-1β, interferon-γ (IFN-γ), and IL-17 were also recognized as inducers of pendrin expression in airway epithelia [20,23,93]. 

Although the exact link between the modification in pendrin expression and airway diseases is still unclear, various mechanisms were proposed, mainly focusing on alterations of the airway surface liquid (ASL) thickness and mucus production. Indeed, following interleukin stimulation (as well as stimulation with allergens or viruses), pendrin is expressed on the apical membrane of bronchial epithelial cells, where it moves Cl^−^ into the cells and secretes HCO_3_^−^ or SCN^−^ in order to regulate ASL thickness, promote mucus production, and contribute to innate defense in the respiratory mucosa [96]. The possible mechanisms leading to this outcome and determining the increase in pendrin expression are described in the subsequent sections.

### 3.1. Molecular Mechanisms for Increased Pendrin Expression

Since both IL-4 and IL-13 are related to the increase in pendrin expression, an in-depth examination of the mechanism involved was performed. As introduced in the previous section, the activation of STAT6 is the main downstream effect of the IL-4/IL-13 signaling cascade. It was, therefore, not surprising that IL-13 stimulation resulted in no increase in pendrin expression in STAT6 knockout mice, unequivocally indicating a contribution of STAT6 in the upregulation of pendrin expression following IL-4/IL-13 stimulation [21]. The situation was similar in an in vitro study aiming to describe the molecular mechanism leading to increased pendrin expression following IL-4/IL-13 stimulation in human lung cells. Indeed, the study showed that human lung cells derived from a lymph node metastasis of a pulmonary mucoepidermoid carcinoma (NCI-H292) had a higher pendrin promoter activity when treated with the two cytokines [22]. The same study showed that the pendrin promoter contained an N_4_ GAS motif, to which the binding of STAT6 was necessary for higher pendrin mRNA expression following Th2 cytokine stimulation in NCI-H292 cells [22]. Later, the presence of a second N_4_ GAS motif, located more than 1600 base pairs upstream with respect to the one previously identified, and about 3400 base pairs 5′ to the pendrin open reading frame (ORF), was reported. Although STAT6 was shown to bind this second N_4_ GAS motif in vitro, the result was not recapitulated in vivo, leading to the conclusion that only the first of the two N_4_ GAS motifs is functional and necessary for interleukin-stimulated increases in pendrin expression [97]. These data strongly suggest that increased promoter activity through STAT6 binding is at least one of the mechanisms via which IL-4 and IL-13 increase pendrin expression in airway epithelia. 

A further role for IL-4 or IL-13 in pendrin upregulation may rely on epigenetics, in particular DNA methylation. DNA methylation refers to the transfer of a methyl group on carbon number 5 of cytosines followed by a guanine (CpG). When a series of closely located CpG sites forming a CpG island is present 5′ of a gene, their methylation leads to the binding of methyl-CpG-binding proteins causing chromatin remodeling and interference with regulatory transcription factors, with transcriptional repression as a general result [98,99,100,101,102]. As such, different levels and patterns of CpG site methylation can determine alterations in gene expression. Studies on the pendrin promoter revealed a CpG island containing 91 CpG sites, located between the previously identified functional N_4_ GAS motif and ORF [103]. In particular, this island was differentially methylated in two different cell lines, NCI-H292 and a clone of human embryonic kidney (HEK) cells constitutively overexpressing STAT6. Compared to the NCI-H292 model, significantly more cells from the HEK model were methylated at the two CpG sites in close proximity to the functional N_4_ GAS motif [103]. The same study also showed that the increase in pendrin mRNA following IL-4 exposure was greater in the NCI-H292 cells, suggesting that basal methylation levels may impact the magnitude of transcriptional response [103]. Further in vitro studies aiming to define this possible correlation demonstrated the ability of IL-4 to cause site-specific demethylation of the two aforementioned CpG sites, thereby prompting the authors to speculate that their demethylation may be a prerequisite for STAT6 binding to the closely located N_4_ GAS motif and eventual changes in pendrin gene expression [104]. This was in line with a previous observation showing that IL-4 caused gene-specific demethylation during monocyte differentiation [105]. In conclusion, these data reveal a new role for Th2-mediated signaling in terms of gene regulation, since IL-4 involvement in CpG site methylation may define the speed and/or magnitude of pendrin mRNA levels.

Other cytokines, such as IL-17 and IL-1β, were proposed as mediators of alternative pathways for increases in pendrin expression. With regards to IL-17, recent studies on mouse models infected with *Bordetella pertussis* demonstrated a toxin-related increase in both pendrin mRNA and protein levels [106]. Further studies in *B. pertussis* infected STAT6 knockout (KO) mice identified this increase as independent from STAT6 but related rather to IL-17A [93]. This was also in accordance with previous data showing increased IL-17 production only in those mice infected with a *B. pertussis* strain able to produce pertussis toxin [107]. In addition, an analysis of the ion transport in well-differentiated human bronchial epithelial cells showed a higher bicarbonate secretion following IL-17 stimulation [108]. Moreover, studies from the same year showed a time-dependent increase in pendrin mRNA and protein expression following stimulation of bronchial epithelial cells with IL-17, together with a correct localization of the exchanger on the apical membrane [30]. Surprisingly, the situation in nasal polyps tissue was different, since neither IL-13 nor IL-17 alone was correlated with an increase in pendrin expression. In cultured nasal epithelial cells, on the other hand, both cytokines were able to upregulate the expression of pendrin when studied singularly and, moreover, showed a synergistic effect when analyzed in combination [92]. The increase in pendrin expression induced by IL-13 and IL-17 alone was greater when the cells were infected with rhinovirus [92]. Of note, IL-13 was shown to be the only cytokine inducing the fully functional form of pendrin, which is glycosylated [92]. The authors suggested that the discrepancy between the ex vivo nasal polyps and the cultured cells in terms of pendrin expression was probably due to the timing of the sample collection, as well as the limit of detection for IL-17. Indeed, even IL-17 quantities below the limit of detection may be sufficient for the synergistic effect with IL-13 leading to increased pendrin expression [92]. IL-17 is one of the main drivers for neutrophil infiltration, which is a typical condition in patients with severe asthma [109]. Put together, these data suggest that pendrin may be maximally expressed in severe asthma, since, in this pathological condition, IL-17, as well as IL-4 and IL-13, are abundant in the airway epithelia. Similarly, the combination of IL-17 and IL-13 may explain the increased pendrin expression seen in COPD, given that both cytokines are also elevated in this disease state [82,110,111]. Studying thiocyanate (SCN^−^) movement in human bronchial epithelial cells, Pedemonte et al. described an increased pendrin mRNA expression following IL-1β treatment [23]. Similarly, Hogmalm et al. showed a higher pendrin expression in the developing lungs of fetal mice expressing human IL-1β under the control of the surfactant protein promoter [112]. In the same study, in vitro measurement of pendrin mRNA and protein expression in differentiated human nasal epithelial (HNE) cells was increased by the co-operation of IL-1β with IL-13. These data point to a further role for IL-1β induced pendrin in inflammatory and infectious diseases in upper and lower airways [92].

### 3.2. Pendrin as a Regulator of the Airway Surface Liquid

**Figure 1:**
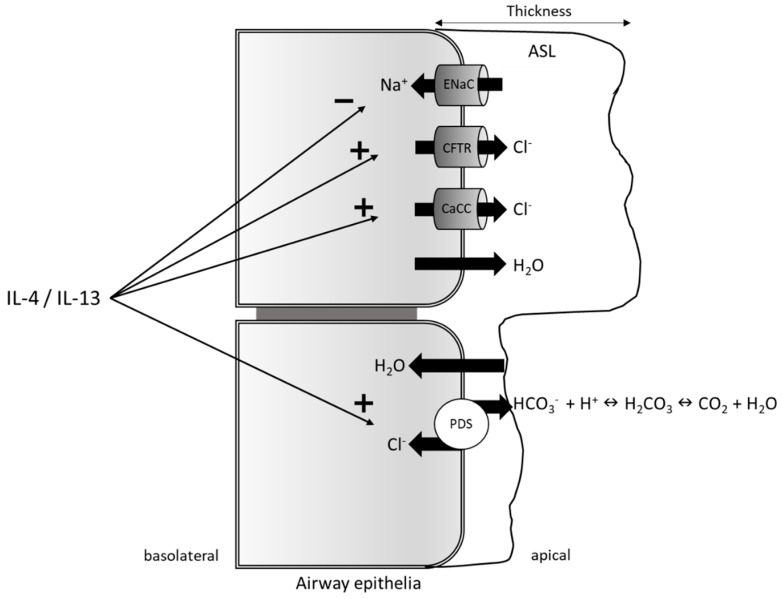
Schematic model for airway surface liquid (ASL) thickness regulation (modified from References [22,96]). In airway epithelial cells, interleukin (IL)-4 and IL-13 increase Cl^−^ secretion acting on expression and/or activity of cystic fibrosis transmembrane conductance regulator (CFTR) and calcium-activated chloride channels (CaCCs) while decreasing Na^+^ reabsorption through epithelium sodium channels (EnaCs). This would result in a higher ion concentration in the lumen, with water following the osmotic gradient and increasing ASL thickness. IL-4 and IL-13, however, increase pendrin expression, thus leading to Cl^−^ reabsorption and HCO_3_^−^ secretion. HCO_3_^−^ is then combined with H^+^ and transformed to CO_2_ and H_2_O by the carbonic anhydrase (CA) enzymes in the lumen, leading to net water reabsorption and eventually decreasing ASL thickness.

The intense release of IL-4 and IL-13 in the airways leads to airway narrowing, pulmonary inflammation, airway hyperresponsiveness (AHR), and increased mucus secretion, all typical features of asthma [31]. In particular, IL-13 is responsible for many of the physiological and structural changes driven by allergic inflammation in various tissues [113]. In the bronchial epithelium, a fundamental role is attributed to the ASL, a thin fluidic layer whose composition and thickness is regulated by several transporters and ion channels, aquaporin (AQP) water channels, salt-sensitive enzymes, and peptide antibiotics [96]. Interestingly, many of these entities deputed to ASL regulation are altered by IL-4 and IL-13 [114]. Both cytokines increase the expression and activity of calcium-activated chloride channels (CaCCs) and the cystic fibrosis transmembrane conductance regulator (CFTR) [18,115,116,117,118], but downregulate the epithelial sodium channel (EnaC) [117]. This action could result in higher Cl^−^ secretion and lower Na^+^ reabsorption, leading to an osmotic gradient which would increase ASL thickness and mucus fluidity, both beneficial effects in the bronchial epithelium of asthmatic patients [96]. However, IL-4 and IL-13 also increase pendrin expression on the apical membrane of airway epithelia, which could lead to the uptake of Cl^−^ in exchange for HCO_3_^−^ [119]. Once in the lumen, HCO_3_^−^ is neutralized to H_2_CO_3_, which is then transformed into H_2_O and CO_2_ by carbonic anhydrases (CA2) [120], leading to a decreased ion concentration. The resulting loss of the osmotic gradient would remove water from the lumen, eventually nullifying the previously described beneficial effects (Figure 1) [22]. First demonstrations of pendrin involvement in the regulation of ASL thickness were described in a report showing that, in pendrin-deficient mice following IL-13 stimulation, the ASL was thicker with respect to wild-type mice [20]. In contrast to this finding, later studies demonstrated that, in human Calu-3 serous airway epithelial cells, pendrin was involved in the majority of the apical HCO_3_^−^ secretion in exchange of Cl^−^, thereby impacting the ASL’s pH, even if it showed a lesser effect on fluid secretion [119]. Trying to further elucidate pendrin’s role in the regulation of the ASL thickness, Lee et al. demonstrated that the ASL layer was thicker in HNE cells from patients with a mutated SLC26A4 compared to controls [121]. These results are particularly intriguing, considering that the ion transporter expression pattern in airway epithelia showed no difference between patients carrying the SLC26A4 mutation with respect to controls, except for a decreased CFTR expression. These findings demonstrate a role for pendrin in ASL volume regulation, even without interleukin stimulation [121]. However, these data are in contrast with the situation described in mouse [20], although such a discrepancy may arise from the difference in the ion transporter expression profile, considering, for instance, that CFTR is barely expressed in mouse airway epithelia. It should also be acknowledged that, as an electroneutral transporter, pendrin alone cannot generate an osmotic gradient, and it is, therefore, not easy to understand its involvement in the ASL regulation. As previously described, under an inflammatory condition, IL-13 induces airway fluid secretion with an increased ASL thickness as a result. In this view, it may not be surprising that the difference in ASL thickness between pendrin-deficient patients and controls is even more prominent following IL-13 treatment. Indeed, in normal airway epithelial cells, increased expression of pendrin following IL-13 treatment compensates for the increased Cl^−^ secretion mediated by the CaCC anoctamin 1 (ANO1), which is also overexpressed in inflammatory conditions [121]. This compensatory mechanism regulates the ASL thickness but is lost in patients with mutated pendrin, resulting in an increased accumulation of ASL [121]. Together, these data reveal a multi-faceted role for pendrin in the regulation of ASL thickness. Indeed, in a normal situation, pendrin may be important for the correct balance in the airway lumen ion composition, while, under inflammatory conditions (IL-4/IL-13 stimuli), it may cause ASL dehydration, leading to airway inflammation and obstruction, thus increasing disease severity.

### 3.3. Pendrin and Mucus Production

Mucus accumulation in the airway would lead to a perfect environment for bacteria to survive and proliferate [122]. Excessive mucus production was initially related to CF, but is also a common feature in bronchial asthma and COPD, and contributes to increased morbidity and mortality in these diseases [123,124,125]. For instance, in bronchial asthma, IL-4 and IL-13 are the leading cause of mucus hypersecretion and goblet cell hyperplasia [126,127,128]. Nakao et al., using a mouse model overexpressing pendrin, demonstrated increases in mucus exudates and Muc5ac protein, one of the major molecules in mucus, in bronchoalveolar lavage (BAL) fluid, together with greater AHR and neutrophilic infiltration [21]. Furthermore, they induced an in vitro production of MUC5AC in NCI-H292 cells with an IL-13-driven increase in pendrin expression [21].

Seshadri et al. showed a similar situation in patients having chronic rhinosinusitis with nasal polyps; indeed, high levels of IL-13 in sinonasal tissues upregulated pendrin expression in nasal polyps of the patients in the study, and this correlated with increased levels of MUC5AC [92]. Another study in the same year demonstrated low expression levels of MUC5AC in the nasal epithelium of patients carrying a non-functional pendrin, and no significant changes in mucin expression were seen even following IL-13 stimulation of the nasal epithelial cells coming from the same patients [121]. The same study also revealed that mutations on the pendrin gene leading to a non-functional transporter did not result in IL-13-driven goblet cell hyperplasia in the nasal epithelia, probably a consequence of the lower number of goblet cells in the epithelia of patients carrying a mutation with respect to the controls [121].

**Figure 2:**
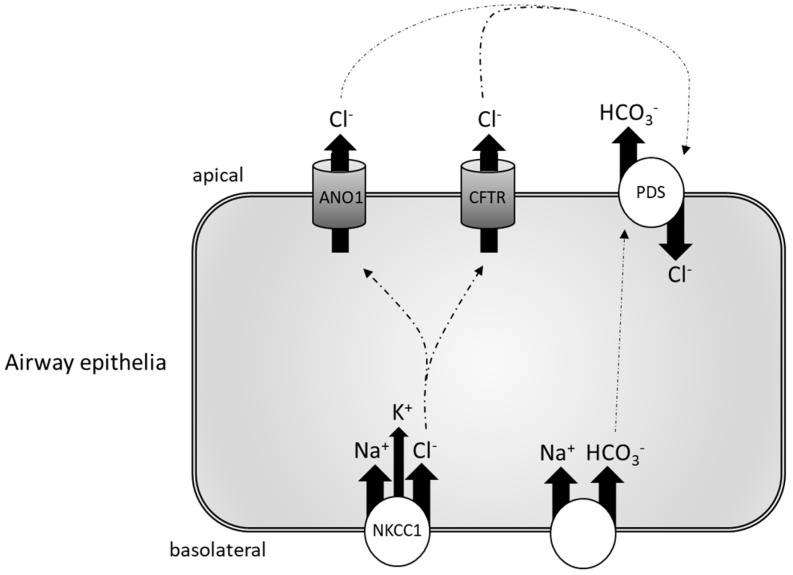
Schematic model for mucus secretion in bronchial epithelial cells (modified from Reference [132]). Na^+^–K^+^–Cl^−^ co-transporter (NKCC1) promotes the basolateral absorption of Cl^−^, which is then secreted into the lumen by CFTR and anoctamin 1 (ANO1), while pendrin exchanges Cl^−^ from the lumen with intracellular HCO_3_^−^. This equilibrium is dysregulated by inflammatory events, with an increased HCO_3_^−^ secretion leading to higher mucus formation. For graphic simplicity, all the transporters are shown in the same cell, although they may be expressed in different cells of the airway epithelium.

The importance of pendrin in mucus release may rely on the fact that bicarbonate secretion is necessary for the correct release and expansion of mucus [129,130,131]. Investigating the effects of prolonged IL-4 stimulation on ion transporters and their correlation with goblet cell hyperplasia, Gorrieri et al. defined a possible model for increased mucus release in bronchial epithelial cells [132]. Indeed, the authors showed an upregulation of the basolateral Na^+^–K^+^–Cl^−^ co-transporter (NKCC1), together with the increased expression of ANO1 and pendrin following 72-h treatment with IL-4. Higher NKCC1 expression would lead to an increased basolateral uptake of Cl^−^, which is then excreted by apical ANO1 and CFTR. Pendrin would then exchange Cl^−^ for HCO_3_^−^, thus resulting in higher bicarbonate levels in the airway lumen, eventually leading to mucus production (Figure 2) [132].

IL-17 is also known as an inducer for MUC5AC in airway [65] and nasal [133] tissues. As previously illustrated, IL-17 acts synergistically with IL-13 to induce pendrin expression in HNE cells [92]. Recent studies showed a significant correlation between pendrin, MUC5AC, and eosinophil infiltration in nasal polyps of eosinophil chronic rhinosinusitis patients; nevertheless, almost half of the studied polyps did not present high levels of pendrin, thus leading to the speculation that IL-17 in combination with Th2 cytokines may be necessary for pendrin expression and mucus production in eosinophil chronic rhinosinusitis [134]. Put together, these data indicate a role for pendrin in mucus production and hyperplasia driven by allergic cytokines in airway epithelia, although the mechanism still needs to be completely elucidated.

### 3.4. Pendrin-Mediated Thiocyanate Secretion

The system formed by lactoperoxidase/H_2_O_2_/SCN^−^, producing the antimicrobial molecule hypothiocyanite (OSCN^−^), is known to be important for the innate defense against bacteria, fungi, and viruses in the airways [135,136]. SCN^−^ is incorporated into the epithelial cells by the Na^+^/I^−^ symporter (NIS)/SLC5A5 located on the basolateral side, and is then actively secreted into the lumen. Here, SCN^−^ reacts with H_2_O_2_ produced by dual oxidase 1 (DUOX1) and 2 (DUOX2) leading to OSCN^−^ production. In particular, this reaction is catalyzed by myeloperoxidases, eosinophil peroxidases, and lactoperoxidases, expressed by neutrophils, eosinophils, and epithelial cells, respectively [137]. Various ion channels and transporters were proposed as mediators of SCN^−^ transport in the lumen, with the main candidate being CFTR, which was shown to be involved in cyclic AMP (cAMP)-dependent SCN^−^ transport [138]. Moreover, CF cells lacking in functional CFTR showed decreased bactericidal activity [139]. Interestingly, Pedemonte et al. described alternative, IL-4-sensitive pathways for SCN^−^ movements. Indeed, they showed that bronchial epithelial cells had a higher transepithelial SCN^−^ flux when treated with IL-4, independent from CFTR and correlated with a strong pendrin upregulation [23]. Intriguingly, the same treatment revealed an upregulation of DUOX1 and DUOX2 expression, leading to a higher H_2_O_2_ production and, in turn, greater OSCN^−^ levels [23,140]. Recent studies from Suzuki et al. revealed a role in the initiation of airway inflammation for the OSCN^−^ produced by the pendrin/DUOX/peroxidase pathway. Indeed, the authors showed an OSCN^—^ mediated activation of NF-κB via protein kinase A (PKA) in airway epithelial cells, which in turn stimulates the production of chemokines and other inflammatory cytokines [141]. Not surprisingly, production of OSCN^−^ mediated by this pathway is increased in mouse models of asthma, as well in some asthma patients, depending on the severity and/or the treatment undertaken [142]. One of the most intriguing outcomes of these studies is the possibility of repositioning antithyroid drugs targeting elements of the OSCN^−^ production machinery as antiasthma agents, as demonstrated, for instance, with studies on the heme peroxidase inhibitors, thus decreasing the economic impact that the research and development of novel drugs would create [137,142]. In conclusion, pendrin-mediated OSCN^−^ production appears to be an innate mechanism for host defense against pathogens in the airways. However, inflammatory conditions leading to increased expression of pendrin, DUOX, and peroxidases would result in an increased pulmonary inflammation and allergy severity.

## 4. Pendrin in the Esophagus

**Figure 3:**
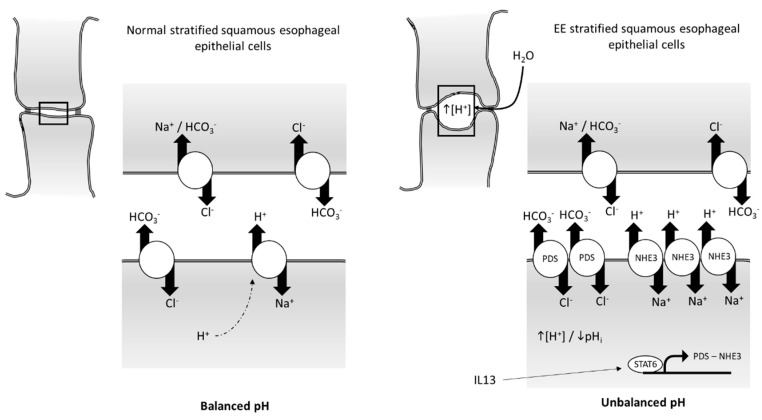
Schematic model for intracellular pH regulatory circuit in normal and eosinophilic esophagitis (EE) stratified squamous esophageal epithelial cells (modified from Reference [152]). In normal epithelium, intracellular pH is kept balanced by the presence of Na^+^-driven Cl^−^/HCO_3_^−^ exchanger (NCBE), Na^+^/H^+^ exchanger (NHE3), and HCO_3_^−^/Cl^−^ exchangers (pendrin (PDS), SLC4A2. And SLC4A8). During IL-13 inflammatory conditions in EE, expression of pendrin and NHE3 is increased, leading to unbalanced pH and water influx causing dilated intercellular spaces (DIS) and epithelium disruption.

While pendrin involvement in airway diseases is well studied, the situation is different in the esophagus, where pendrin involvement is less clear, and mostly related to EE, a food allergen-induced Th2-driven inflammatory disease, characterized by eosinophilic infiltration and upregulation of IL-5 and IL-13 release [37,143,144]. In particular, IL-13 is a central mediator of the disease [36,145]. In esophageal epithelial cells, cytosolic H^+^ concentration may increase due to cell metabolism or via back diffusion from refluxed gastric acid. In both cases, this would lead to a reduction of intracellular pH to acidic levels that cells are not able to balance with their buffer capacity. In order to avoid cell damage from a decreased cytosolic pH, under normal conditions, esophageal epithelial cells express a series of ion transporters that function to remove excess intracellular H^+^ and restore pH. Such transporters include members of the Na^+^-dependent Cl^−^/HCO_3_^−^ and Na^+^/H^+^ exchanger families, lactate-H^+^ transporters, and the vacuolar H^+^-ATPase [146,147,148,149,150,151]. Na^+^ is, therefore, used to directly remove intracellular H^+^ or to exchange intracellular Cl^−^ for extracellular HCO_3_^−^ to balance pH. If these combined actions result in a pH above 7.4, an Na^+^-independent Cl^−^/HCO_3_^−^ exchanger acts to lower intracellular pH by exchanging extracellular Cl^−^ with intracellular excess of HCO_3_^−^ [146]. Pendrin expression was shown to be increased in both the murine IL-13-induced transcriptome and human EE biopsy samples (by 6.125- and 8.25-fold, respectively) [29]. A similar situation was seen for most of the other genes involved in the intracellular pH regulatory circuit, including the Na^+^/H^+^ exchanger SLC9A3, other Cl^−^/HCO_3_^−^ exchangers (SLC4A2, SLC4A8), and CA2. In particular, Zeng et al. proposed a model that, in normal stratified squamous esophageal epithelial cells, the intracellular pH is kept balanced by the combined action of the Na^+^-driven Cl^−^/HCO_3_^−^ exchanger (NCBE), the Na^+^/H^+^ exchanger (SLC9A3 or NHE3), and the Cl^−^/HCO_3_^−^ exchangers (pendrin, SLC4A2, and SLC4A8) [152]. In EE stratified squamous epithelial cells, the author suggested that a STAT6-mediated, IL-13 driven increase in NHE3 and Cl^−^/HCO_3_^−^ exchangers (with pendrin as the most dysregulated between them) would cause an unbalance in the intracellular pH and higher osmotic force between cells, leading to dilated intercellular spaces (DIS) and epithelial disruption (Figure 3) [152].

## 5. The Therapeutic Potential of Pendrin in Airway and Esophageal Inflammatory Diseases

When speaking about airway inflammatory diseases, there are several differences in terms of therapies. For instance, asthma treatment relies on inhaled corticosteroids as anti-inflammatory agents and on long-acting β-adrenergic agonists for bronchodilation, while the most popular therapy for COPD is the use of anti-cholinergics as bronchodilators [96]. In the treatment of esophageal inflammatory diseases, swallowed corticosteroids are considered the main therapy for EE patients, together with dietary control, especially when proton pump inhibitors are not responsive [153]. Although these therapies are effective in the control of the aforementioned diseases, there are some patient populations, for example, severe asthmatics, where the effectiveness of these treatments remains low. A secondary stream of studies focused on counteracting inflammations by blocking cytokine signaling, as happened, for example, with the development of Th2-cytokine inhibitors as a therapy for asthma [154]. Despite some promising results in animal models, the efficacy of these inhibitors was not the same in humans, although, in some cases, they showed some encouraging effects in the treatment of specific patients subtypes such as corticosteroid-resistant or severe eosinophilic asthmatics [155,156]. In a similar way, mixed results were also obtained with this strategy for EE treatment; an anti-IL13 therapy in a randomized controlled trial showed moderate histologic improvement in EE [157], while another study aiming to evaluate the efficacy of the anti-immunoglobulin E (IgE) omalizumab did not show any histological or endoscopic improvement [158]. In recent years, there was an increased understanding that asthma and COPD [154], as well as rhinosinusitis [159] and EE [160], are not simply inflammatory, but also epithelial diseases, since defects in barrier function may increase allergens or pathogens penetration and wall remodeling [161,162]. In this view, pendrin may elicit a role in the pathogenesis of these inflammatory diseases, impairing ASL thickness, mucus production, or intracellular pH regulation, as described before. Several studies demonstrated increased pendrin expression and activity following Th2-cytokine stimulation. However, the question arises whether the inflammation is at the basis of pendrin overexpression or whether the expression of this exchanger is already upregulated in the patients, and cytokine release is only creating a positive feedback loop. This question is even more intriguing considering that, although IL-4 and IL-13 signaling do not have the same contribution in the pathogenesis of asthma and COPD, in both diseases, pendrin is similarly overexpressed. The fact that overexpression of pendrin alone drives higher mucus formation, AHR, and respiratory neutrophilic infiltration in human airway epithelial cells may support the idea that increased pendrin expression is causing epithelial remodeling even prior to cytokine release, identifying pendrin as a possible primary therapeutic target [21]. For clinical purposes, it would be extremely important to confirm the in vitro and animal model data identifying pendrin’s role in airway epithelia directly in human. The first attempt in this direction was done by Madeo et al. who tried to correlate Pendred syndrome patients with asthma resistance, showing that none of the patients had asthma symptoms, although a low number of participants caused the study not to reach statistical significance [163]. More recently, Lee et al. analyzed pendrin in primary cell cultures derived from HNE cells demonstrating that results from previous studies in mouse models could be translated to HNE [121,164]. Since specific pendrin inhibition in the respiratory or esophageal epithelium may represent a novel strategy for therapy of inflammatory diseases, such as asthma, COPD, or EE, in the past years, the effort in the discovery of such blockers was high. In 2016, Haggie et al. identified selective pendrin blockers in the classes of the tetrahydropyrazolopyridine and pyrazolothiophenesulfonamide compounds, and, in particular, the molecule named PDS_inh_-A01 showed the highest specificity and was able to increase ASL hydration in IL-13-stimulated human bronchial epithelial cells from healthy subjects and patients with CF [165]. More intriguing would be the possibility of using inhibitors that are already on the clinical market. One example could be the anti-inflammatory drug niflumic acid, which is a well-established inhibitor of ion transport, and in particular a non-selective pendrin blocker [166,167]. Studies on asthma animal models already revealed that this drug is able to reduce the IL-13-mediated development of the disease, including features like AHR, goblet cell hyperplasia, and eosinophil degranulation and accumulation [168,169]. Similarly, tenidap, an anti-rheumatic, anti-inflammatory drug known to inhibit ion transport, was shown to block pendrin activity following expression in HEK cells [166]. Although the effects of this drug on airway or esophageal inflammatory diseases are not known, it was already tested in a few clinical trials for rheumatic arthritis [170,171]; it would be, therefore, interesting to investigate possible uses of this drug in the treatment of respiratory or esophageal distresses.

## 6. Conclusions

In this review, we provided a description of the IL-mediated pendrin transcriptional regulation and the consequent dysregulation of various molecular mechanisms during inflammatory response in airway and esophageal epithelia. In the airways, increased pendrin expression is linked to diseases such as asthma, COPD, rhinitis, chronic rhinosinusitis, and pertussis, where it dysregulates the ASL thickness, induces mucus production, and initiates the inflammatory process via OSCN^−^. In the esophageal epithelium, pendrin may be involved in the pathogenesis of EE by impacting the subtle equilibrium for pH regulation in the esophageal epithelial cells. Put together, these studies highlighted the importance of pendrin in response to inflammatory diseases, thus identifying it as a possible target for the treatment of these pathologic manifestations.

## Abbreviations

AHR	Airway hyperresponsiveness
ANO1	Anoctamin 1
AQP	Aquaporin
ASL	Airway surface liquid
BAL	Bronchoalveolar lavage
CA2	Carbonic anhydrase
CACCs	Calcium-activated chloride channels
C/EBP	CCAAT/enhancer-binding protein
CBP	CREB-binding protein
CCL	Chemokine (C-C motif) ligand
CD4^+^	Cluster of differentiation 4^+^
CF	Cystic fibrosis
CFTR	Cystic fibrosis transmembrane conductance regulator
COPD	Chronic obstructive pulmonary disease
CREB	cAMP response element-binding
CXCL	Chemokine (C-X-C motif) ligand
DIS	Dilated intercellular spaces
DUOX	Dual oxidase
EE	Eosinophilic esophagitis
EnaC	Epithelial sodium channel
GAS	Gamma activated sequence
G-CSF	Granulocyte colony-stimulating factor
HEK	Human embryonic kidney
HNE	Human nasal epithelial
IFN-γ	Interferon-γ
IL	Interleukin
JAK	Janus kinase
KO	Knockout
MAPK	Mitogen-activated protein kinase
NCBE	Na^+^-driven Cl^−^/HCO_3_^−^ exchanger
NcoA-1	Steroid nuclear receptor co-activator 1
NF-κB	Nuclear factor kappa-light-chain-enhancer of activated B cells
NHE3	Na^+^/H^+^ exchanger
NIS	Na^+^/I^+^ transporter
NKCC1	Na^+^-K^+^-Cl^−^ co-transporter
NKT	Natural killer T cells
ORF	Open reading frame
OSCN^−^	Hypothiocyanite
PI3K	Phosphoinositide-3-kinase
SCN^−^	Thiocyanate
SH2	Src homology 2
STAS	Sulfate transporter anti-sigma factor antagonist domain
STAT	Signal transducer and activator of transcription
TGF	Transforming growth factor
Th2	T helper cell type 2
TMD	Transmembrane domain
TNF	Tumor necrosis factor
